# Formulation and *In Vitro *Characterization of Xanthan Gum-Based Sustained Release Matrix Tables of Isosorbide-5- Mononitrate

**Published:** 2010

**Authors:** Rajat Kar, Snehamayee Mohapatra, Satyabrata Bhanja, Debjyoti Das, Bhaktibhusan Barik

**Affiliations:** a *Jeypore College of Pharmacy, Jeypore, Koraput, India. *; b *UDPS, Utkal University, Vani Vihar, Bhubaneswar, India.*

**Keywords:** Isosorbide, Xanthan gum, Sustained release, Matrix tablet, Release rate

## Abstract

In the present investigation an attempt has been made to increase therapeutic efficacy, to reduce frequency of administration and to improve patient compliance by developing a sustained release matrix tablets of isosorbide-5-mononitrate. Sustained release matrix tablets of isosorbide-5-mononitrate were developed by using different drug: polymer ratios, such in F1 (1:0.75), F2 (1:1), F3 (1:1.5), F4 (1:1.75) and F6 (1:2). Xanthan gum was used as matrix former and microcrystalline cellulose as diluent. All the lubricated formulations were compressed, using 8mm flat faced punches. Compressed tablets were evaluated for uniformity of weight, content of active ingredient, friability, hardness, thickness, in vitro dissolution study using basket method and swelling index. Each formulation showed compliance with pharmacopoeial standards. Among all formulations, F5 showed a greater sustained release pattern of drug over a 12 h period with 92.12% of drug being released. The kinetic studies showed that drug release follows the Higuchi model (r^2^ =0.9851). Korsemeyer and Peppas equation gave an n-value of 0.4566, which was close to 0.5, indicating that drug release follows the Fickian diffusion. Thus, xanthan gum can be used as an effective matrix former to extend the release of isosorbide-5-mononitrate. No significant difference was observed in the dissolution profile of optimized formulation, using basket and paddle apparatus.

## Introduction

Increased compliance and expense involved in marketing of new drug entities has focused greater attention on development of sustained release or controlled release drug delivery systems (1). Matrix systems are the most popular method among innumerable methods used in the development of controlled release formulations. Hydrophilic polymeric matrix systems are widely used in controlled drug delivery, since they make it easier to achieve a desirable drug release profile, are cost effective and have broad FDA acceptance (2). 

Xanthan gum is a high molecular weight extracellular polysaccharide, produced in commercial scale from the fermentation of gram negative bacterium *Xanthomanas campestries*. It is a hydrophilic polymer, which until recently had a limited use as thickening, suspending and emulsifying agent in water based systems. It is now being used in gum based sustained release tablet matrices. Xanthan gum not only retards drug release, but can also provide time independent release kinetics with added advantage of compatibility and inertness. Release of soluble drugs from this biopolymer occurs mainly through diffusion, whereas sparingly soluble or insoluble drugs are released as a result of matrix erosion. It is also recommended for use in both acidic and alkaline media (3). Xanthan gum has been evaluated as a controlled release formulation for model drugs, including theophylline (4), cefalexime (5) and indomethacin (6).

Isosorbide-5-mononitrate (ISMN, a polyol ester of nitric acid) is a nitro vasodilator, which is most widely used for the treatment of angina pectoris. The half life of ISMN is 4.9 h and its usual time interval for administration regimen is 6 to 8 h. Hence, in order to reduce the frequency of administration and to improve patient compliance, a sustained release formulation of ISMN is desirable. ISMN is freely soluble in water, hence judicious selection of the release retarding excipients is necessary to achieve a constant in vivo input rate of the drug.

In the present study, various hydrophilic matrix systems were designed by taking different concentrations of xanthan gum, with a fixed drug concentration.

## Experimental

**Materials**

Isosorbide-5-mononitrate was a gift sample from Mecleods Pharmaceuticals, Mumbai. Pharmacopoeial grade xanthan (USP / NF) gum with a 1% aqueous solution viscosity of 1350 cps at 25°C and particle size less than 14.28 μm was obtained from Loba Chemicals, Mumbai.All the other materials used were of analytical grade, procured from commercial sources.

**Methods**

*Preparation of sustained release matrix tablets*

Tablets were prepared by the direct compression method, using different drug: polymer ratios, of 1:0.75, 1:1, 1:1.25, 1:1.5, 1:1.75 and 1:2, etc. as per given in Table 1. Xanthan gum was used as a matrix forming agent while microcrystalline cellulose was used as diluent. All the ingredients, except glidant and lubricant, were passed through a 100-mesh sieve, weighed and blended. The lubricated formulations were compressed by direct compression, using 8-mm flat faced punches (8 station rotary tablet machine, Rimek Minipress-I, India).

**Table 1:** Components of sustained release matrix tablets All batches contain 19% diluted ISMN (ISMN: lactose ratio is 1:1), 1.9% magnesium stearate, 0.47% aerosil

**Ingredients **	**Formulations**					
	**F1**	**F2**	**F3**	**F4**	**F5**	**F6**
Xanthan gum	14.2%	19%	23.8%	28.57%	33.3%	38%
Microcrystalline cellulose	44.76%	40%	35.23%	30.47%	25.71%	20.9%

*Evaluation of physical properties*

All the prepared matrix tablets were evaluated for uniformity of weight and drug content, based on the Indian pharmacopoeia method. Friability was determined, using a Roche friabilator. Hardness was measured using a Pfizer hardness tester. The thickness was measured by varnier caliper (7-9). 

*In vitro dissolution test*

In vitro drug release was performed using a USP apparatus I (rotary basket), with 500 mL of dissolution medium maintained at 37±1°C for 12 h, at 50 rpm. 0.1N HCl (pH 1.2) was used as the dissolution medium for the first 2 h followed by pH 7.2 phosphate buffer for the next 10 h. Samples were withdrawn at 0.5, 2, 4, 8 and 12 h intervals, respectively. The amounts of dissolved drug were then determined spectrophotometrically (UV/Vis Shimadzu spectrophotometer model-1601) at 223 and 221 nm, using filtered portions of the samples. The drug released at any time interval was obtained by calculating the mean cumulative percent of drug release belonging to six tablets from each formulation.

*Drug release kinetics*

To study the release kinetics, data obtained form in-vitro drug release studies were plotted in various kinetic models: zero order (equation 1), as the cumulative percentage of drug release vs. time, first order (equation 2), as the log of the amount of drug remaining to be released vs. time and Higuchi model (equation 3), as the cumulative percentage of drug release vs. square root of time.

C = K_0_ t                     (equation 1)

where K_0_ is the zero order rate constant. A graph of concentration vs. time would yield a straight line, with a slope equal to K_0_ and intercept the origin of the axes (10). 

Log C = Log C_0_ – K_1_t/2.303                      (equation 2) 

where C_0_ is the initial concentration of drug and, K_1_ is the first order rate constant (11). 

Q = K_h_t^1/2 ^                      (equation 3) 

where K_h_ is the constant reflecting design variables of the system and t is the time in hours. Hence, drug release is proportional to the reciprocal of time (12). 

In order to evaluate the extent drug release, with a change in the surface area and diameter of the particles/tablets, data were also plotted using the Hixson-Crowell cube root law (equation 4) 

Q_0_^1/3^ – Q_t_^1/3^ = K_hc_ . t                      (equation 4) 

where Q_t_ is the amount of drug to be released in time t, Q_0_ is the initial amount of drug in the tablet and K_hc_ is the rate constant for Hixson- Crowell rate equation (13). In here the cube root of the percentage of drug remaining in the matrix vs. time is plotted. 

*Mechanism of drug release*

To evaluate the mechanism of drug release from ISMN sustained release tablets, data of drug release was plotted in Korsmeyer et al’s equation (equation 5), as the log of cumulative % of drug released vs. log time, and the exponent ‘n’ value was calculated through the slope of the straight line.

M_t_ / M_∞_ = Kt^n ^                      (equation 5) 

where M_t_ / M_∞_ are the fractional solute released, t is the release time, K is the kinetic constant of drug-polymer system and ‘n’ is an exponent that characterizes the mechanism of drug release (14). 

For a cylindrical matrix tablets, if the exponent n = 0.45, then the drug release mechanism is Fickian diffusion, and if 0.45 < n< 0.89 then it is non-Fickian diffusion. An exponent value of 0.89 is indicative of case II transport or typical zero order release (15). 

*Comparative evaluation of dissolution profiles*

To evaluate and compare the dissolution profile of each batch of tablets, whenever necessary, the similarity factor (f_2_), which may be defined as follows: 

f_ 2_ = 50 log { [1+1/n Σ wt. (R_t_ – T_t_) ^2^] ^- 0.5^ x 100 } 

where n is the number of withdrawal points, wt optional weight factor, R_t_ is reference assay at time t and T_t_ is test assay at time t. Factor f_2_ is inversely proportional to the averaged squared difference between the two profiles and measures the closeness between the two profiles. A f_2_ value between 50 to 100 suggests that the dissolution profiles are similar, and a value of 100 suggests that the test and reference profiles are identical. As the value become smaller, the dissimilarity between release profiles increases (16). 

*Stability studies*

The optimized formulation was subjected to stability testing at 40 ± 2°C and 75 ± 5% RH, for a period of six months. After each month the tablet samples were analyzed for physical attributes and in vitro drug release profile (17). 

*Swelling behaviors of sustained release matrix tablets*

The extend of swelling was measured in terms of the percentage weight gained by the tablet. The swelling behavior of all the formulations prepared were studied. One tablet from each formulation was placed in a petri dish containing pH 7.2 phosphate buffer. After 1 h, the tablet was withdrawn, soaked with tissue paper and reweighed. Then, after every 2 h, weights of the tablets were noted and the process was continued till the end of 12 h, the percentage weight gained by the tablet was calculated using the formula: S.I. = {(M_t_ – M_0_) / M_0_} × 100, where, S.I. = swelling index, Mt = weight of the tablet at time‘t’ and M_0_ = weight of tablet at time t = 0 (18). 

*Statistical analysis*

All statistical calculations were performed using the Sigma Stat 3.5 demo version software. Data were expressed as mean ± SD and analyzed using one way analysis of variance (ANOVA) followed by post hoc method (Tukey test) as per the requirement. Differences were considered statistically significant at P < 0.05. 

## Results and Discussion

The main objective of this study was to enhance therapeutic performance of ISMN by developing matrix tablets. Xanthan gum was selected as the matrix former in this investigation. 

The composition of various tablet formulations prepared have been shown in Table 1. 

The physical attributes of the prepared tablets were found to be satisfactory. Typical tablet defects such as capping, chipping and picking were not observed. Results of rhe other physical evaluations like weight variation, thickness, hardness, and friability of the prepared tablets were analyzed in order to the examine their influence on the extent of drug release from the tablet matrices prepared. All these properties compiled with the official limit, as shown in Table 2. 

**Table 2:** Physical properties of ISMN (Isosorbide-5-mononitrate) matrix tablets

**Formulation **	**Weight variation (n = 20) **	Drug content* (%)	**Hardness (Kg/cm** ^2^ **) (n = 10) **	**Friability **	**Thickness (mm) (n = 10) **
F1	209.5 ± 3.1	99.6 ± 0.45	6.90 ± 0.12	0.31	4.20 ± 0.04
F2	208.7 ± 3.1	99.2 ± 0.21	5.60 ± 0.21	0.12	4.23 ± 0.01
F3	209.2 ± 3.0	98.5 ± 0.045	5.40 ± 0.02	0.39	4.24 ± 0.02
F4	210.1 ± 5.3	100.4 ± 0.63	4.60 ± 0.26	0.34	4.21 ± 0.01
F5	209.0 ± 3.5	99.3 ± 0.20	7.25 ± 0.98	0.40	4.20 ± 0.00
F6	209.0 ± 3.9	100.3 ± 0.12	6.77 ± 0.96	0.50	4.22 ± 0.02

*Mean of triplicate with SD

**Figure 1:**
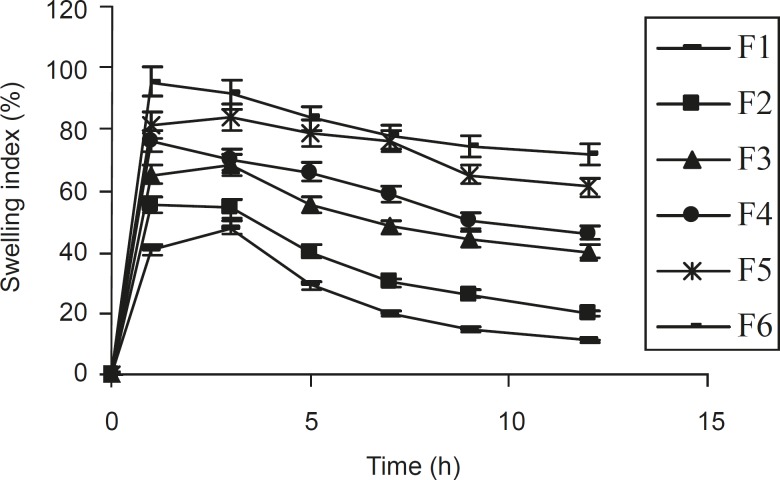
Relationship between the swelling index and time, obtained for different ISMN tablets formulationsprepared (n = 6, mean ± SD).

From the study on swelling index (Figure 1), it was observed that as the contact time increases, swelling index also increased, since the weight gained by the tablet was increased proportionally with the rate of hydration up to 3 h. Later on, it decreased gradually due to the dissolution of the outermost gelled layer of test tablet into the dissolution medium. A direct relationship was observed between the swelling index and gum concentration. As the gum concentration increases, the swelling index was also increased. Furthermore, it was observed that the cumulative percentage of drug release decreased with a increasing concentration of gum and swelling index. 

Ideally, an extended release tablets should release the required quantity with predetermined kinetic pattern in order to maintain an effective drug plasma concentration. To achieve this, the tablet should be formulated so that it releases the drug in a predetermined and reproducible manner. By considering the biopharmaceutics and pharmacokinetic profile of the drug, one can determine the required amount of drug release from the tablets (19). 

**Figure 2:**
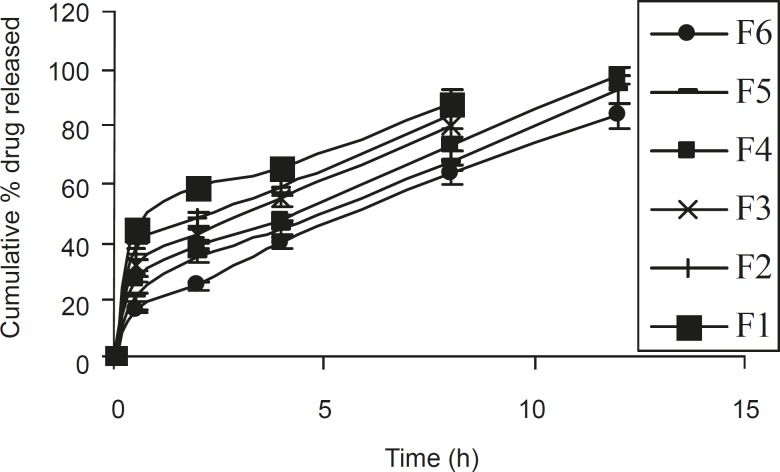
Release profiles of different ISMN sustained release tablet formulation**s **(n = 6, mean ± SD).

Based on dissolution profiles of various tablet formulation, an inverse relationship between the amount of gum present and the released rate of the medicament was observed by increasing the concentration of gum within the formulations F1 to F6 from 14.2 % to 38 % w/w, a slower rate and significant (P < 0.05) decrease in the amount of drug release from different tablet formulation was noted. In formulations F1, F2, and F3 (which contained 14.2%, 19% and 23.8% w/w polymer), 88.46%, 83.92% and 80.8% of drug content was released in within 8 h. However, in formulations F4, F5 and F6, which contained 28.57%, 33.3% and 38% w/w of polymer, 97.54%, 92.78 and 83.77% of drug content was released in 12 h (Figure 2). This gradual slow release was due to the formation of a thick gel structure that delayed drug release from tablet matrix, where hydration of individual xanthan gum particles results in extensive swelling. As a result of the rheological nature of the hydrated matrix, the swollen particles would coalesce. This results in a continuous viscoelastic matrix that fills the interstices, maintaining the integrity of the tablet and retarding further penetration of the dissolution medium (20). 

The kinetic studies showed that formulations F1 and F2 had the highest regression (r^2^) values for first order, kinetics, implying only a small effect of polymer concentration on the release rate of drug. When kinetic studies of the rest of formulations (F3, F4, F5 and F6) were considered, the highest r^2 ^values of 0.9705, 0.9799, 0.9851 and 0.9812, respectively, were found for the Higuchi model (Equation 3). This described that the release rate of drug from swellable matrix tablets are propotional to the square root of time. Comparing the similarity factors (f_2_) of different formulations with the theoretical release profiles, the f_2_ value of formulation F3 was found to be insignificant (45.616), since the release rate of drug is greater than the expected value. However, the other formulations had significant value of 58.26, 74.6 and 58.94, respectively. Among them formulation F5 was the best one based on its highest f_2_ value. 

In formulation F5, the correlation coefficient obtained, indicated a good fit with the Higuchi model (Table 3), suggesting that diffusion is the predominant mechanism of drug release in this hydrophilic matrix formulation. When the tablet comes into contact with the dissolution medium, it would take up water and swells to form a gel layer around the matrix. 

**Table 3:** Mathematical modeling and drug release kinetics of the prepared ISMN tablet formulation.

Formulation	r^2^				f_2_ value	n-value
	Zero order	First order	Higuchi model	Korsmeyer model		
F1	0.724	0.937	0.914	0.95	32.29	0.233
F2	0.803	0.952	0.948	0.927	39.14	0.273
F3	0.855	0.964	0.97	0.942	45.62	0.321
F4	0.932	0.886	0.979	0.945	58.26	0.456
F5	0.948	0.931	0.985	0.975	74.66	0.456
F6	0.971	0.98	0.981	0.967	58.94	0.233

**Figure 3:**
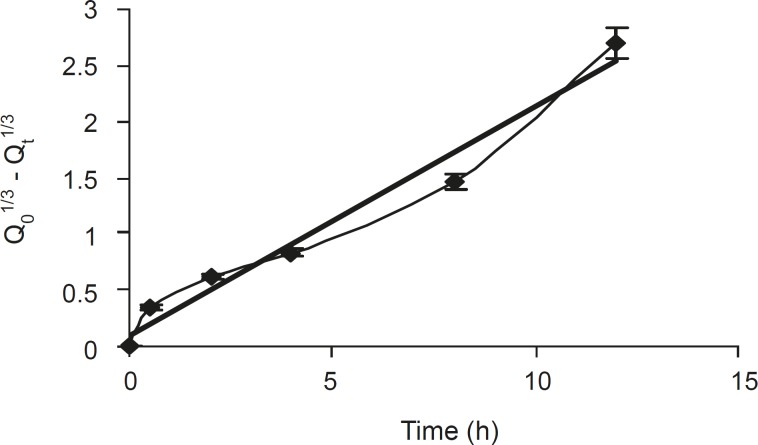
Hixon Crowell release profiles of formulation F5 (n = 6, mean ± SD).

The dissolved drug would then diffuse out of the matrix and enter the dissolution medium. Rao et al (21) had proposed that for highly water soluble drugs, the diffusion and swelling fronts are similar and rate of drug release is determined by diffusion of the drug from the gel, which in turn is dependent on gel thickness and poorly soluble drugs dependent on erosion of the matrix, predominantly. The dissolution data were also plotted in terms of the Hixon-Crowell cube root law (Equation 4) of formulation F5. The compliance of this formulation to the equation indicated a change in surface area and diameter of tablets, due to the progressive dissolution of the matrix as a function of time (Figure 3). 

**Figure 4:**
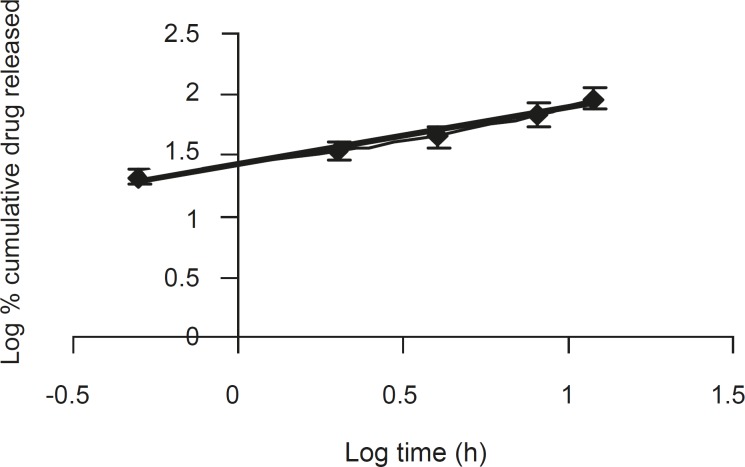
A log-log plot of the korsemeyer- peppas equation for formulation F5 (n = 6, mean ± SD).

The corresponding plot (log cumulative % of drug released vs. log time) for Korsemeyer- Peppas equation (Figure 4) indicated a good linearity (r^2^ = 0.975) for the F5 formulation F5. The release exponent ‘n’ was 0.4566, which is very close to the value expected forthe the Fickian diffusion. 

**Figure 5:**
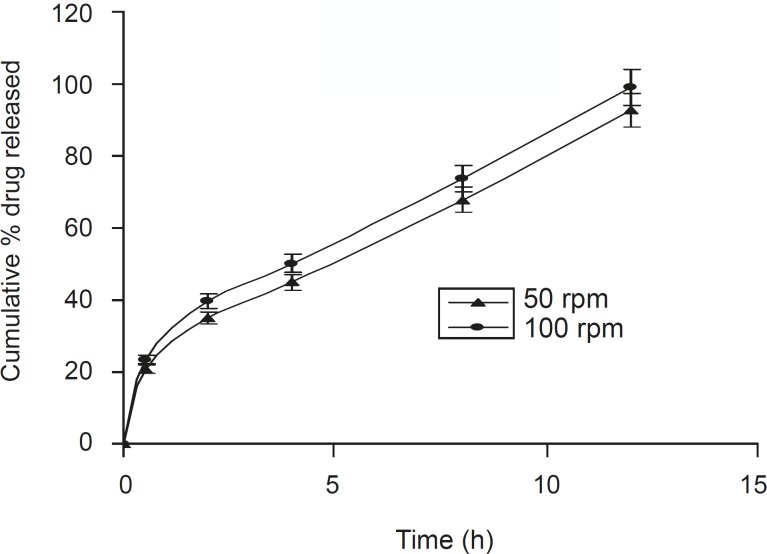
Effect of the speed of rotation on dissolution profile of the optimized formulation F5 (n = 6, mean ± SD).

A positive influence of speed of rotation during the in vitro drug release study was noted (Figure 5). As the stirring speed was increased, the thickness of the hydrated gelatinous layer surrounding the intact tablet core was noticeably decreased, resulting in a more rapid release of drug from the tablet matrix. The was also reported by Talukdar et al (22). 

**Figure 6:**
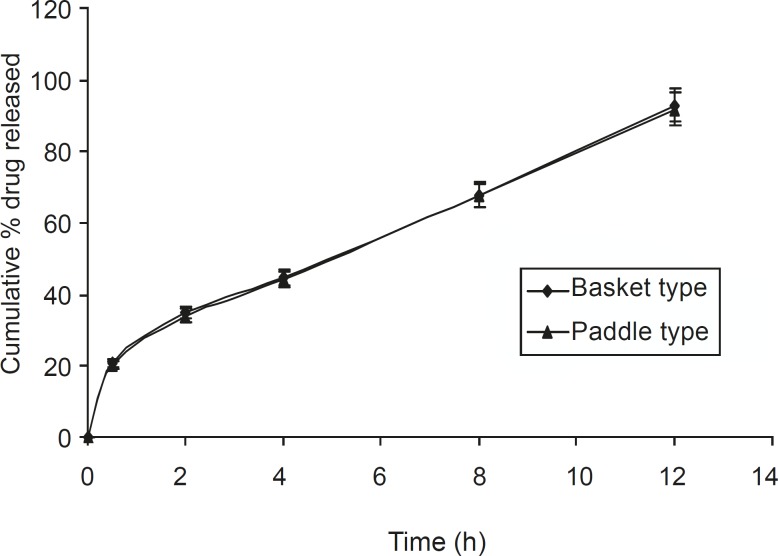
Comparative dissolution study of the optimized formulation F5, using the basket and paddle apparatus (n = 6, mean ± SD).

Dissolution studies were also carried out using the paddle method, keeping the rotation speed 

at 50 rpm (Figure 6). It was observed that tablets present within the buffer solution, settled down to the bottom of the flask. It swelled considerably to release drug from the matrix. There was no significant difference between the basket and paddle method, under the same experimental condition (P > 0.05), when data were analyzed using one way ANOVA (Sigma Stat 3.5 software).

Stability study revealed that there was no significant change (P > 0.05) in hardness, friability, drug content and in vitro dissolution profiles of the optimized formulation F5. Thus, this formulation was stable at different conditions of temperature and humidity.

## Conclusion

In conclusion, the results of the present study demonstrated that xanthan gum could be a successful hydrophilic polymer for the formulation of sustained release matrix tablets of Isosorbide-5-mononitrate. In vitro dissolution studies indicated a sustained release pattern throughout the 12 h study period, which was compatible with theoretical release profile. Hence xanthan gum based matrix tablets seem to have a desirable sustained pattern of drug release, in order to reduce the dosing frequency.
